# Expanding the reach of the Quitline by engaging volunteers to market it in hospitals and shopping venues – a pilot study

**DOI:** 10.1186/s12971-015-0040-0

**Published:** 2015-06-10

**Authors:** Fadi Hammal, Alyssa Chappell, Katherine Pohoreski, Barry A. Finegan

**Affiliations:** Department of Anesthesiology and Pain Medicine, University of Alberta, Edmonton, Canada

**Keywords:** Smoking, Cessation, Promotion, Quitline

## Abstract

**Background:**

In Canada, although there are periodic media campaigns to raise awareness of Quitlines, these services are underused. We sought to determine if a dedicated kiosk, similar to that used in the retail industry but staffed by volunteers trained in smoking cessation techniques, would be effective method to enhance Quitline reach.

**Methods:**

We located a kiosk in the foyer of two hospitals and in two shopping malls in Edmonton, Canada between Feb/2012 and July/2014. The cessation intervention was based on the 5 A's approach. Outcome was assessed by number of visits to the kiosk and referral rates to the Quitline. A cross sectional survey among small sample of visitors was used for evaluation. Descriptive statistics were used to summarize visitors’ data.

**Results:**

Of 1091 kiosk visitors, 53.3 % were current smokers, of whom 93.3 % indicated a willingness to quit. Of these, 32.1 % requested a Quitline referral at the time of the kiosk visit. Referral requests to the Quitline were greater when the kiosk was located in the non-hospital setting 39.1 % compared to 31.1 % in hospitals (*P* = 0.2). Referrals from the kiosk represented 6 % of total referrals received by the provincial Quitline during the study period. Following referral the Quitline was able to reach 50 % of those referred, of those, 17 % refused to proceed. At seven month follow up 30 day abstinence rate was 3.8 % of smokers who wished quit. Visitors agreed that the kiosk design was interesting (89.3 %) and increased their knowledge about tobacco and cessation options (88.8 %) and encouraged them to take action to quit (85.7 %).

**Conclusions:**

A “volunteer manned kiosk” can increase awareness of smoking cessation resources in the community and increase referral rates to Quitline services.

## Background

Progress has been made in reducing the prevalence of smoking in Canada as evidenced by the latest Canadian Tobacco Use Monitoring Survey (CTUMS) data from 2012, which details an overall smoking prevalence rate in the country of 16 % [1, 2]. Comparable findings were reported in USA, where smoking prevalence among adults has decreased to 17.8 % in 2013 [3]. Nevertheless, it is clear that despite this success there remains a “health inequalities gap”, with individuals of both lower income and educational attainment being more likely to be current smokers, engage in daily smoking and consume more cigarettes than the rest of the population [4, 5]. This is strikingly reflected in the CTUMS data, where the daily smoking rate among those employed in the education/government/social/ religious sectors was reported to be 9.2 % whereas a rate of 32.5 % was reported for those individuals working in the manufacturing sector [6]. Reaching the latter group of smokers has proven to be particularly difficult and may require a reorientation of public health efforts toward personalized delivery of prevention and cessation information and face-to-face engagement of the target population [7].

Treatment of smoking-related illness consumes considerable acute healthcare hospital resources [8], with approximately one third of all patients aged between 45 and 74 years being smokers or former smokers [9]. Paradoxically, these facts, although concerning, present an invaluable opportunity for smoking cessation intervention and education. Smokers attending hospital facilities appear to be ready for such engagement. A recent study of smokers attending outpatient surgical assessment clinics found that ~ 60 % were willing to participate in a brief or intensive smoking cessation intervention program offered at the time of the clinic visit [10]. Furthermore, staff, visitors and patients faced with smoke-free grounds policies in hospitals appear to be in need of on-site education about tobacco addiction and information about cessation services [11, 12]. In Canada, there is uneven delivery of such services in tertiary care environments even for patients [12].

Available data from market research indicate the potential of kiosks to increase the awareness of the product or service offered, capitalize on foot traffic, and stimulate impulse-buying consumption [13]. The kiosk industry continues to grow worldwide, especially in North America, contributing to about 10 % of shopping malls revenue [14]. Written educational materials remain the preferred way to provide patients with information regardless of their effectiveness [15]. Supplementing written materials with verbal advice has added benefits over using printed materials alone and is more effective in improving patients’ knowledge and satisfaction [16, 17]. Combining these approaches we have developed the concept of a “manned” kiosk dedicated to promoting smoking cessation, education and tobacco use prevention, supplementing printed materials with verbal advice from qualified personnel.

The primary objective of our pilot study was to determine if locating a volunteers-operated kiosk in the common area of hospitals and non-hospital settings would be effective in encouraging smokers to seek assistance to quit by requesting referral to the provincial telephone Quitline. Our secondary objective was to check the outcome of the Quitline contact with those referrals.

## Methods

### Design

A cross sectional survey was conducted among a small sample of visitors to evaluate the acceptance of this approach to service. The potential effectiveness of a smoking cessation kiosk located in hospitals public spaces and in shopping centers was determined by the number of visitors and the rate of referral to the local smoking cessation Quitline.

### Sample and setting

The kiosk, branded to support a province-wide initiative Tobacco Free Futures [18], was located near the main entrances of acute care hospital and community hospital, and in non-hospital settings in Alberta, Canada. Areas of higher traffic flow were selected for the kiosk service as this significantly impacts the reach of the kiosk. The kiosk welcomed all visitors and operated for an average of 2.5 h shifts starting in Feb/2012 and ending in July 2014. Individuals who were 18 years and older and able to read and write English were asked if they were willing to complete a self-administered evaluation survey.

**Fig. 1:**
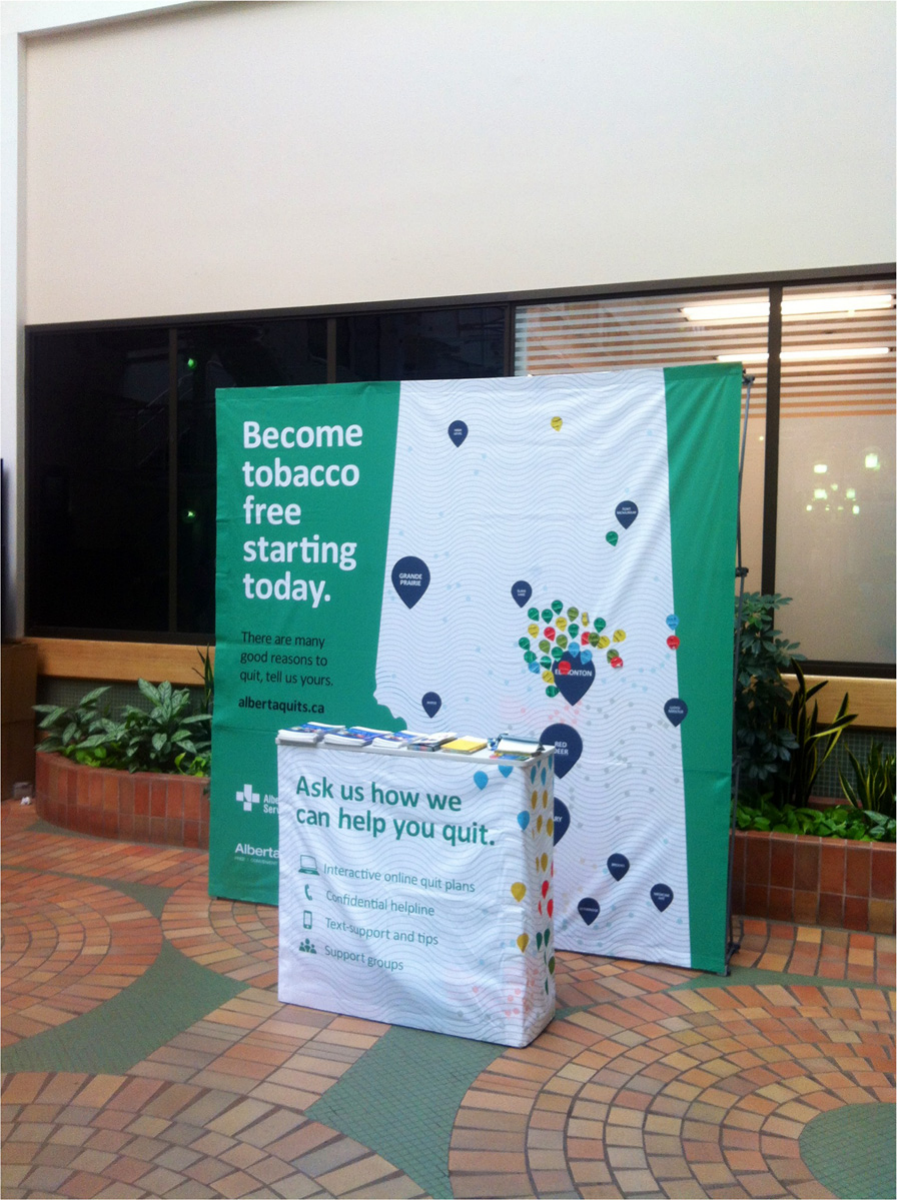
The Kiosk

The kiosk was designed to promote Alberta Quits, a provincial smoking cessation program. The backdrop image is a map of the province, whereby visitors can place success stickers to display their quit decision (Fig. 1).

### Procedures

The kiosk was created to be operated by volunteers trained in tobacco reduction/cessation techniques. We promoted our volunteer program with minimal advertising within the health science buildings and the School of Public Health at the University of Alberta, as well as the University’s online volunteer resource database. Students who contacted us expressing interest were asked to provide two references, and after an interview were required to obtain a police record check, sign a volunteer registration form, waiver of liability and choose a shift time and location based on their availability. Each volunteer had to commit to a minimum of about 5 h/month to the project. Volunteers were trained to provide brief intervention to visitors at the kiosk, specifically to individuals who smoked and/or wanted to help someone quit. Training included either: 1) a two-day Tobacco Reduction and Cessation (TRAC) training intended for tobacco reduction counselors and healthcare professionals and provided by Alberta Health Services (AHS) or 2) a condensed version of the AHS-TRAC for those who were not available to attend the full length course. Additionally, all volunteers were required to attend a session which outlined kiosk practices. A volunteer coordinator looked after scheduling and preparing the kiosk with sufficient resource materials and data collection sheets. The procedure for operating the kiosk is built upon the 5As model for behavioral change. Nevertheless, as the mandate of the kiosk was to “sell health”, there was great freedom in engaging in optimistic, non-judgmental conversation with the visitors and encouraging them to use Quitline services and smoking cessation resources.

All Quitline referrals were submitted to the coordinator following a shift and were faxed immediately Quitline service. Visitors Log data were entered to a database. An evaluation survey collected in the first days of operation in order to gain feedback about the kiosk experience. Volunteer shifts are logged in terms of the date, location, volunteer name, time and duration of shift, number of visitors and referrals, and referral rate.

### Intervention

Individuals voluntarily approached the kiosk at their own discretion, and staff would engage in a non-judgemental and therapeutic conversation using the 5A’s model for behavioural change;[19] by: 1) asking visitors about tobacco use; 2) advising visitors that quitting smoking is the best thing smokers can do for their overall health; 3) assessing visitors’ interest in quitting and discussing previous quit attempts; 4) assisting visitors by providing smoking cessation information and describing a sample quit plan, and 5) arranging for follow-up through referral to the Quitline. The Quitline is a free and confidential service offering counselling and support to smokers wishing to quit [20]. Those wishing to access the Quitline service were referred directly at the kiosk. For a referral, the individual’s name, phone number and the best way to be contacted was collected and faxed to the Quitline, which contacted the client with 48 h of receiving the request.

Those who did not self-identify as smokers were asked if they wanted smoking cessation material on behalf of a smoker. A third hand assessment was completed: 1) how often the non-present person smoked; 2) any previous quit attempts and 3) if they had openly voiced that they had wanted to quit. Visitors were offered appropriate materials as requested dealing with second hand smoke, third hand smoke, advice on how to discuss tobacco use with their children, and/or material to assist others to stop tobacco use. Printed resources distributed at the kiosk were ordered online through Alberta Quits [20].

### Measures

The self-administered evaluation survey collected the participant’s general demographic information, present smoking status and smoking habits, attitude toward quitting using standardised (0 to 10) Likert scale questions about interest in quitting, importance of quitting and confidence in ability to quit, about the reason in hospital and the motivation to approach the kiosk, and their perceptions about their visit to the kiosk using a 8-point Likert scale to rate different statements. A visitor log was kept to gain information about those who visited the kiosk. The log included information on the location, date and time the person visited the kiosk, their gender, smoking status, the material dispensed and referral services provided. A summary of outcome of referrals was provided by the Quitline including the ability to reach and the self-reported quit rates at 7 month follow-up. The study and all data collection instruments were approved by the ethics review committee at the University of Alberta. Informed consent was obtained from all participants.

### Data analysis

Data were summarized using descriptive statistics. Categorical variables were reported using frequencies, while continuous data were analyzed using means and standard deviations, comparison between hospitals and non-hospitals settings was performed using cross tabulation. The Statistical Package for the Social Sciences (SPSS, Version 19.0, IBM, Armonk, NY, USA) was used for data management and statistical analyses.

## Results

The kiosk was successfully operated in both health and non-health settings where there were an opportunity to reach a large number of people who require cessation help. In its 80 operating days (72 in the hospitals setting, 8 in non-hospitals setting) and from February, 2012 to July, 2014, a total of 1091 persons (60.5 % female) visited the kiosk. The total operation time for the kiosk was about 200 h with average of 5.5 visitors per hour of operation. Of these, 53.3 % were current smokers and the majority of these (93.3 %) indicated that they wanted to quit smoking. Of non-smoking visitors 77.5 % wanted to help someone they knew quit smoking. A fax referral to the Quitline was requested by 17.5 % of all visitors to the kiosk, either for themselves (174 referrals, 32.1 % of smoking visitors) or for someone close to them (17 referrals, 4.3 % of non-smoking visitors). This could be translated into average of one referral to Quitline for every hour of operation (Table 1).

**Table 1:** Comparison between Healthcare setting vs. non-healthcare setting

	Healthcare setting	Non healthcare setting	Total		
	n/N (%)	n/N (%)	n/N (%)	*χ* ^2^	*P*-value
Sex					
Female	602/965 (62.4)	58/126 (46.0)	660/1091 (60.5)	12.47	0.001
Male	363/965 (37.6)	68/126 (54.0)	431/1091 (39.5)		
Smoking Status					
Smoker	505/965 (52.3)	76/126 (60.3)	581/1091 (53.3)	4.11	0.12
Non-smoker	447/965 (46.3)	50/126 (39.7)	497/1091 (45.6)		
No answer	13/965 (1.3)	-	13/1091 (1.2)		
Smoker interested in quitting	473/505 (93.7)	69/76 (90.8)	542/581 (93.3)	0.87	0.33
Non-smoker wanted to help another quit	352/447 (78.7)	38/50 (76.0)	390/497 (78.5)	0.20	0.72
Fax Referral filled					
All visitors	164/965 (17.0)	27/126 (21.4)	191/1091 (17.5)	1.52	0.21
Smokers interested in quitting	147/473 (31.1)	27/69 (39.1)	174/542 (32.1)	1.79	0.21
Non-smokers wanted to help someone quit	17/352 (4.8)	0/38 (−)	17/390 (4.4)	1.91	0.39
Visitor got education materials	833/965 (86.3)	106/126 (84.1)	939/1091 (86.1)	0.45	0.50

Of kiosk visitors, 68 individuals agreed to participate in the kiosk evaluation survey. Overall, 41.2 % of evaluation study participants were current smokers and 20.6 % were former smokers. About 86 % of current smokers had made quit attempts in the past and reported that they were currently thinking of quitting or taking actions to quit. Of smokers who completed the evaluation survey, 14.3 % were patients, 32.1 % were family members of a patient or visitors, 7.1 % were hospital employees, and the remaining (46.4 %) were in non-hospital setting (Table 2).

**Table 2:** Kiosk evaluation survey

Kiosk evaluation survey								
	Smokers (*n* = 28)				Non-smokers (*n* = 40)			
Reason in hospital								
Patient (%)	14.3				22.5			
Family of patient (%)	25.0				7.5			
Visitor (%)	7.1				10.0			
Employee at hospital (%)	7.1				45.0			
Other or non-hospital setting (%)	46.4				15.0			
Why approached the kiosk								
Interested in quitting (%)	96.4				5.6			
Want to help someone quit (%)	-				72.2			
Interest sake (%)	3.6				11.1			
Time to spare (%)	-				11.1			
Statements about kiosk	Strongly agree	Agree	Neutral	Disagree	Strongly agree	Agree	Neutral	Disagree
Information increased my knowledge (%)	44.4	44.4	11.1	-	42.5	42.5	12.5	2.5
Information influenced my point of view (%)	35.7	60.7	3.6	-	38.5	28.2	28.2	5.1
Information encouraged action (%)	46.4	39.3	10.7	3.6	46.4	28.6	17.9	7.1
Design is interesting (%)	50.0	39.3	10.7	-	60.5	36.8	2.6	-
Approach is effective (%)	71.4	28.6	-	-	55.0	42.5	2.5	-
Message is relevant to me (%)	53.6	42.9	-	-	59.5	37.8	2.7	-
Would share the information (%)	53.6	42.9	3.6	-	59.0	41.0	-	-

Approximately 96 % of smokers who participated in the survey approached the kiosk because of an interest in quitting, while only 4.0 % approached the kiosk just for curiosity or because they were otherwise unoccupied. Smoking participants agreed or strongly agreed that the kiosk design was interesting (89.3 %), effective (99.6 %), provided information that was relevant to them (96.5 %), increased their knowledge about tobacco related issues (88.8 %), influenced their point of view (96.4 %) and that the visit encouraged them to take action (85.7 %) (Table 2).

Referrals from the kiosk represented (191/3246, 5.9 %) of total referrals received by the Quitline from the whole province during the study period. The Quitline was able to reach 50 % of the referred smokers and of those 17 % refused the service. At 7 month –follow up, the Quitline was able to reach (31/79, 39.2 %) of smokers who accepted the service. At 7 month –follow up, both 7 and 30 days point prevalence abstinence from any tobacco products for total smokers reached was (3/95, 3.6 %), and for smokers who accepted the service was (3/79, 3.8 %). The reported 7 and 30 days point prevalence abstinence rates for smokers responded to 7 month–follow up was (3/28,10.7 %) (Table 3).

**Table 3:** Quitline statistics summary

	n/N	%
Quitline ability to reach		
Yes	79/191^a^	41.3
Yes, Refused Service	16/191	8.4
No	96/191	50.3
Smokers reached at 7 month callback		
Yes	28/79^b^	35.4
Yes, Refused Service	3/79	3.8
No	48/79	60.8
Any tobacco products use in the past 7 days at 7 month follow-up?		
No	3/28^c^	10.7
Yes	25/28	89.3
Any tobacco products use in the past 30 days at 7 month follow-up?		
No	3/28	10.7
Yes	25/28	89.3

^a^Total referrals

^b^Smokers who accepted the service at the initial contact

^c^Smokers who responded to 7 month follow-up

## Discussion

Knowledge gained from this first report on the use of an innovative approach to offer on-site brief smoking cessation intervention and referral in the hospital and non-hospital setting indicates that the kiosk was able to; 1) successfully catch visitors’ attention, 2) encourage them to explore what the kiosk was offering 3) provide them with education and information, and 4) refer the interested individuals to the community Quitline.

Currently, almost all hospitals in Canada and USA have adopted a campus smoke free policy, however, implementing such policies is challenging and compliance requires providing patients and visitors who smoke with different support options, including health promotion and education, pharmacotherapies, and cessation assistance [12]. The motivation to trial the kiosk concept in hospitals and non-hospital settings was sparked by commercial market research data detailing the potential of sales kiosks to increase product/service awareness, capitalize on foot traffic and stimulate impulse-buying consumption [21]. Our kiosk was aimed to market and *sell* one “free” health related product – the referral service. The higher number of days of operation in healthcare setting was mainly due the difficulty of access to non-healthcare setting due to logistical and financial barriers.

Smokers who visited the kiosk were interested in quitting, highly rated the importance of quitting but had less confidence in their ability to quit and most have tried to quit in the past. Brief advice from a healthcare professional increases the likelihood that a smoker will quit, particularly if the intervention is intense and unhurried [22, 23]. However, in reality physicians and other healthcare professionals are not always willing or well prepared to deliver the appropriate interventions [24, 25] and frequently cited limited time and inadequate knowledge as the key factors for their behaviour [26, 27]. The kiosk approach represents an opportunity to supplement healthcare professionals’ roles, increases the smokers’ exposure to smoking cessation resources and offers an alternative opportunity for individuals to consider quitting, with immediate assistance for ongoing support by referral to the quitline.

Quitlines have the potential to provide cessation counseling to many individuals and minimize the time and cost required for such an intervention [28]. Quitlines are effective, however, only 1-2 % of tobacco users access this resource annually [29, 30]. Several strategies have been proposed to increase referral rates including integrating referral to the Quitline into routine care [31]. Despite the potential benefit, the referral rate from physicians is low. For example, only 3.5 % of physicians referred patients to Quitline services after a direct mailing campaign to increase referral rates in North Carolina in 2010 [32]. Public marketing campaigns, including TV and other media options, are found to be effective in increasing Quitline call volume but this effect is transient [33]. In our study, using the kiosk generated significant traffic to the Quitline (17.5 % referral rates to the local Quitline among all kiosk visitors and 32.1 % referral rate among smokers interested in quitting). These rates of proactive referral are encouraging, giving the fact that the kiosk was not part of a marketing campaign but simply an effort to provide cessation counselling on an opportunistic basis without any predetermined inclusion criteria and the evidence that both active (as in this case) and passive referral can result in similar cessation rates [34, 35].

The low quit rates at seven month follow-up compared with reported quit rates from other Quitline evaluation studies [36] could be related to several factors including the reliance on behavioural counselling without providing any pharmacotherapy, the time spent with smokers, or the frequency of contact them. However evaluating the reach and outcomes of an existing Quitline service was not the purpose of this study.

The interest in investigating the benefit of using volunteers in smoking cessation initiatives in real world healthcare setting is increasing [37]. The large number of both smokers and non-smokers that visited the kiosk in the short period of time and the fact that referrals from a single kiosk that operated for about 200 h depending on volunteers with minimal costs provided 6 % of the total referrals received by the Quitline indicates that this approach is an effective health promotion strategy to enhance the reach of smoking cessation services.

### Limitations

This is a pilot study and the first insight on the use of this approach in hospitals and non-hospital settings. Although the preliminary results about the kiosk’s effectiveness are encouraging, evaluating the long-term feasibility and cost-effectiveness of the kiosk in increasing referral rates to the Quitline requires longer implementation and follow-up evaluation plans. One of the study limitations is the low number of participants in the evaluation survey, as our purpose was to provide service in a real practice setting we found in the first few days of operation that visitors were reluctant to fill both the quitline referral form and the evaluation survey, and we had to choose what we saw best for smokers which was the referral form. Another limitation for our study is our limited ability to follow-up on the outcomes of the referral results and to validate if the smoker quit successfully.

## Conclusions

Using an innovate approach for health promotion was positively received by the target population and was considered to be presenting relevant information that changed the users’ point of view, encouraging them to take action. Implementing smoking cessation intervention programs in a hospital setting requires providing hospital staff with support in education and training, as well as addressing smoking amongst staff and providing them with access to appropriate treatments including pharmacotherapy and counselling.

Our program “volunteer manned kiosk” represents an opportunity to supplement the role of healthcare professionals, to increase the smokers’ exposure to smoking cessation resources and to facilitate the provision of direct patient counselling on an opportunistic basis and increase referral rates to Quitline services. Current findings encourage conducting long term trials for both impact and economic evaluation of the “manned kiosk in hospitals”.

## References

[1] Health Canada. Canadian Tobacco Use Monitoring Survey (CTUMS) 2012. 2012. http://www.hc-sc.gc.ca/hc-ps/tobac-tabac/research-recherche/stat/ctums-esutc_2012-eng.php

[2] Reid J, Hammond D, Rynard V, *et al.* Tobacco Use in Canada : Patterns and Trends. Waterloo, ON: 2015. http://www.tobaccoreport.ca/2014/TobaccoUseinCanada_2014.pdf

[3] Centers for Disease Control and Prevention (CDC (2014). Current Cigarette Smoking Among Adults - United States, 2005–2013. MMWR Morbid Mortal Wkly Rep.

[4] King BA, Dube SR, Tynan MA (2012). Current tobacco use among adults in the United States: findings from the National Adult Tobacco Survey. Am J Public Health.

[5] Reid JL, Hammond D, Driezen P (2010). Socio-economic status and smoking in Canada, 1999–2006: has there been any progress on disparities in tobacco use?. Can J Publ Heal Can sante publique.

[6] Health Canada. Canadian Tobacco Use Monitoring Survey (CTUMS) 2011 Supplementary Tables. 2011; 2012.http://www.hc-sc.gc.ca/hc-ps/tobac-tabac/research-recherche/stat/_ctums-esutc_2011/ann-eng.php

[7] Harkins C, Shaw R, Gillies M (2010). Overcoming barriers to engaging socio-economically disadvantaged populations in CHD primary prevention: a qualitative study. BMC Public Health.

[8] Baliunas D, Patra J, Rehm J (2007). Smoking-attributable morbidity: acute care hospital diagnoses and days of treatment in Canada, 2002. BMC Public Health.

[9] Wilkins K, Shields M, Rotermann M (2009). Smokers’ use of acute care hospitals–a prospective study. Health Rep.

[10] Sachs R, Wild TC, Thomas L (2012). Smoking cessation interventions in the pre-admission clinic: assessing two approaches. Can J Anaesth.

[11] Lawn S (2011). Habit or addiction: the critical tension in deciding who should enforce hospital smoke-free policies. CMAJ.

[12] Schultz AS, Finegan B, Nykiforuk CI (2011). A qualitative investigation of smoke-free policies on hospital property. CMAJ.

[13] Krishen AS, Bui M, Peter PC (2010). Retail kiosks: how regret and variety influence consumption. Int J Retail Distrib Manag.

[14] Runyan R, Kim J, Baker J (2012). The mall as bazaar : How kiosks influence consumer shopping behaviour. J Mark Manag.

[15] Ellins J, Coulter A. How engaged are people in their health care ? Findings of a national telephone survey. Oxford: 2005. http://www.health.org.uk/public/cms/75/76/313/3834/How engaged are people in their healthcare full report.pdf?realName=vqk1xh.pdf

[16] Ellins J. MS. Supporting patients to make informed choices in primary care: what works? Birmingham: 2009. http://www.birmingham.ac.uk/Documents/college-social-sciences/social-policy/HSMC/publications/PolicyPapers/Policy-paper-4.pdf

[17] Johnson A, Sandford J, Tyndall J (2005). Written and verbal information versus verbal information only for patients being discharged from acute hospital settings to home (Review). Health Educ Res.

[18] Alberta Health Services. Tobacco Free Futures Guidelines. 2014. http://www.albertaquits.ca/helping-others-quit/healthcare-providers/tobacco-free-futures

[19] Fiore MC, Bailey WC, Cohen SJ (2000). Treating Tobacco Use and Dependence. Clinical Practice Guideline.

[20] Alberta Health Services. Alberta Quits Helpline. 2014. http://www.albertaquits.ca/

[21] Krishen A, Bui M, Peter P (2010). Kiosk retailing environments: Exploring the role of regret and variety on consumer behavior. Int J Retail Distrib Manag.

[22] Fiore MC, Jaen CR, Baker TB (2008). Treating tobacco use and dependence 2008 update.

[23] Stead LF, Bergson G, Lancaster T (2008). Physician advice for smoking cessation. Cochrane Database Syst Rev.

[24] Raupach T, Merker J, Hasenfuss G (2011). Knowledge gaps about smoking cessation in hospitalized patients and their doctors. Eur J Cardiovasc Prev Rehabil.

[25] Schultz AS, Bottorff JL, Johnson JL (2006). An ethnographic study of tobacco control in hospital settings. Tob Control.

[26] Smith PM, Sellick SM, Brink P (2009). Brief smoking cessation interventions by family physicians in northwestern Ontario rural hospitals. Can J Rural Med.

[27] Vogt F, Hall S, Marteau TM (2005). General practitioners’ and family physicians’ negative beliefs and attitudes towards discussing smoking cessation with patients: a systematic review. Addiction.

[28] Vidrine DJ, Vidrine JI (2011). Active vs passive recruitment to quitline studies: public health implications. J Natl Cancer Inst.

[29] Stead LF, Perera R, Lancaster T (2006). Telephone counselling for smoking cessation. Cochrane Database Syst Rev.

[30] Stead LF, Perera R, Lancaster T (2007). A systematic review of interventions for smokers who contact quitlines. Tob Control.

[31] Kirst M, Schwartz R. Promoting a smokers’ quitline in Ontario, Canada: an evaluation of an academic detailing approach. Health Promot Int Published Online First: 2013. doi:10.1093/heapro/dat040 10.1093/heapro/dat040 23766445

[32] Mathew M, Goldstein AO, Kramer KD (2010). Evaluation of a direct mailing campaign to increase physician awareness and utilization of a quitline fax referral service. J Health Commun.

[33] Hurd AL, Augustson EM, Backinger CL (2007). Impact of national ABC promotion on 1-800-QUIT-NOW. Am J Health Promot.

[34] Tzelepis F, Paul CL, Wiggers J (2011). A randomised controlled trial of proactive telephone counselling on cold-called smokers’ cessation rates. Tob Control.

[35] Nohlert E, Ohrvik J, Helgason AR (2014). Effectiveness of proactive and reactive services at the Swedish National Tobacco Quitline in a randomized trial. Tob Induc Dis.

[36] North American Quitline Consortium. Tobacco Cessation Quitlines A Good Investment to Save Lives, Decrease Direct Medical Costs and Increase Productivity. 2009. http://c.ymcdn.com/sites/www.naquitline.org/resource/resmgr/docs/naqc_issuepaper_tobaccocessa.pdf

[37] Duffy SA, Ewing LA, Louzon SA (2015). Evaluation and costs of volunteer telephone cessation follow-up counseling for Veteran smokers discharged from inpatient units: a quasi-experimental, mixed methods study. Tob Induc Dis.

